# Prenatal Diagnosis of an Isolated Persistent Left Superior Vena Cava With Absent Right Superior Vena Cava: A Case Report

**DOI:** 10.7759/cureus.115622

**Published:** 2026-09-01

**Authors:** Yushi Abe, Kohsei Tanaka, Satoru Ikenoue, Miori Ogawa, Satoru Shiraishi

**Affiliations:** 1 Department of Obstetrics and Gynecology, Nasu Red Cross Hospital, Tochigi, JPN; 2 Department of Obstetrics and Gynecology, Keio University School of Medicine, Tokyo, JPN; 3 Department of Pediatrics, Nasu Red Cross Hospital, Tochigi, JPN

**Keywords:** absent right superior vena cava, congenital heart defect, fetal ultrasound, persistent left superior vena cava, prenatal diagnosis, right ventricular outflow tract, venous anomaly

## Abstract

Persistent left superior vena cava (PLSVC) with absent right superior vena cava (RSVC) is a rare congenital venous anomaly resulting from the failure of the left anterior cardinal vein to regress. Although often asymptomatic in isolated cases, prenatal diagnosis has clinical benefits, including the exclusion of coexisting congenital heart disease (CHD) and awareness of possible complications during central venous access or pacemaker implantation.

We report the case of a 34-year-old G2P1 woman who underwent a fetal echocardiography at 34 weeks of gestation. Examination of the four-chamber view (4CV) revealed an enlarged coronary sinus adjacent to the left atrium, suggesting a PLSVC. Subsequent assessment of the right ventricular outflow tract (RVOT) and three-vessel view (3VV) confirmed the absence of the RSVC and presence of a persistent left superior vena cava (LSVC). Color and spectral Doppler imaging of the aortic arch and isthmus showed no narrowing, ruling out coarctation of the aorta. The pregnancy progressed uneventfully, and the patient had a spontaneous vaginal delivery at 38 weeks, resulting in a healthy newborn. Postnatal evaluations, including echocardiography, confirmed PLSVC draining into a dilated coronary sinus, with no additional cardiac anomalies identified. Although truly isolated, PLSVC typically does not require special delivery planning or postnatal intervention; prenatal recognition allows clinicians to ensure thorough fetal cardiac evaluation, offer appropriate counseling, and plan for potential challenges with right-sided central venous access in the future.

## Introduction

Persistent left superior vena cava (PLSVC) is the most common anomaly of the thoracic systemic venous return, occurring in approximately 0.3% of the general population [1,2]. It results from the embryologic failure of the left anterior cardinal vein to regress, leading to a left-sided superior vena cava (LSVC) that typically drains into the right atrium via the coronary sinus. In over 90% of cases, a PLSVC coexists with a normal right superior vena cava (RSVC) and is hemodynamically inconsequential [3]. However, in rare instances (estimated in 0.07%-0.13% of patients with congenital heart disease (CHD)), the RSVC is absent, leaving the PLSVC as the sole systemic venous return from the upper body [3,4]. This combination - sometimes termed an “isolated” PLSVC (in the sense of absence of the RSVC) - is an uncommon variant of systemic venous anatomy. In such cases, venous drainage from the head and right arm is rerouted via the left brachiocephalic (innominate) vein into the PLSVC. The anomaly generally remains asymptomatic because venous return still reaches the right atrium, but it can be associated with other malformations or clinical considerations.

Several studies have highlighted the importance of recognizing a PLSVC, especially in the prenatal setting [1,5]. PLSVC is the most frequent systemic venous anomaly found in fetuses and newborns with CHD, reported in about 3%-8% of patients with CHD [1,5]. In fetal echocardiography series, the detection of a PLSVC should prompt a thorough search for additional anomalies, as it is often associated with cardiac defects or situs abnormalities, such as heterotaxy (left or right atrial isomerism) [1,5]. In contrast, when a PLSVC is identified in an otherwise normal fetal heart (with a normally present RSVC and no other CHD), it is often an isolated finding with little hemodynamic impact [6]. Prenatal identification of a PLSVC is still clinically significant, as it warrants a detailed fetal anatomic survey and consideration of karyotyping due to potential associations with extracardiac anomalies or genetic syndromes [5,7]. Nevertheless, if no other abnormalities are present, an isolated PLSVC carries a good prognosis [6,8].

Absent RSVC accompanying a PLSVC further elevates the rarity and clinical interest of the anomaly. PLSVC with absent RSVC has been reported both in the context of complex CHD (e.g., as part of heterotaxy syndrome with atrioventricular septal defects) and, less commonly, in patients with otherwise normal cardiac anatomy [3]. A review of 121 cases by Bartram et al. noted additional cardiovascular malformations in 46% of patients with absent RSVC and PLSVC, confirming that nearly half of such cases are part of broader CHD [3]. The same review reported that rhythm disturbances, such as sinus node dysfunction or atrioventricular block, occurred in about one-third of cases, usually in older patients - potentially related to the long-term dilation of the coronary sinus and associated conduction tissue anomalies [3]. Thus, although an isolated PLSVC with or without an RSVC is often benign, its presence - particularly when the RSVC is absent - should prompt clinicians to consider associated anomalies and to plan for management implications.

## Case presentation

A 34-year-old G2P1 woman was initially noted to have a suspected fetal cardiac abnormality on a routine second-trimester morphology ultrasound at an outside facility and was referred to a tertiary center. She was subsequently transferred to our hospital for delivery. At 34 weeks of gestation, she underwent a routine fetal ultrasound. The four-chamber view (4CV) revealed an enlarged coronary sinus adjacent to the left atrium, raising suspicion of a PLSVC. Further evaluation of the right ventricular outflow tract (RVOT) and three-vessel view (3VV) confirmed the absence of the expected RSVC and the presence of a vascular structure on the left side of the mediastinum, consistent with an LSVC (Figure 1). In addition, no extracardiac abnormalities were identified on ultrasound. Color Doppler evaluation of the aortic arch and isthmus demonstrated normal antegrade flow, with no significant narrowing (Figure 2), effectively excluding coarctation of the aorta. Given the isolated nature of the PLSVC (with absent RSVC) and the otherwise normal fetal survey, the pregnancy was managed expectantly.

**Figure 1:**
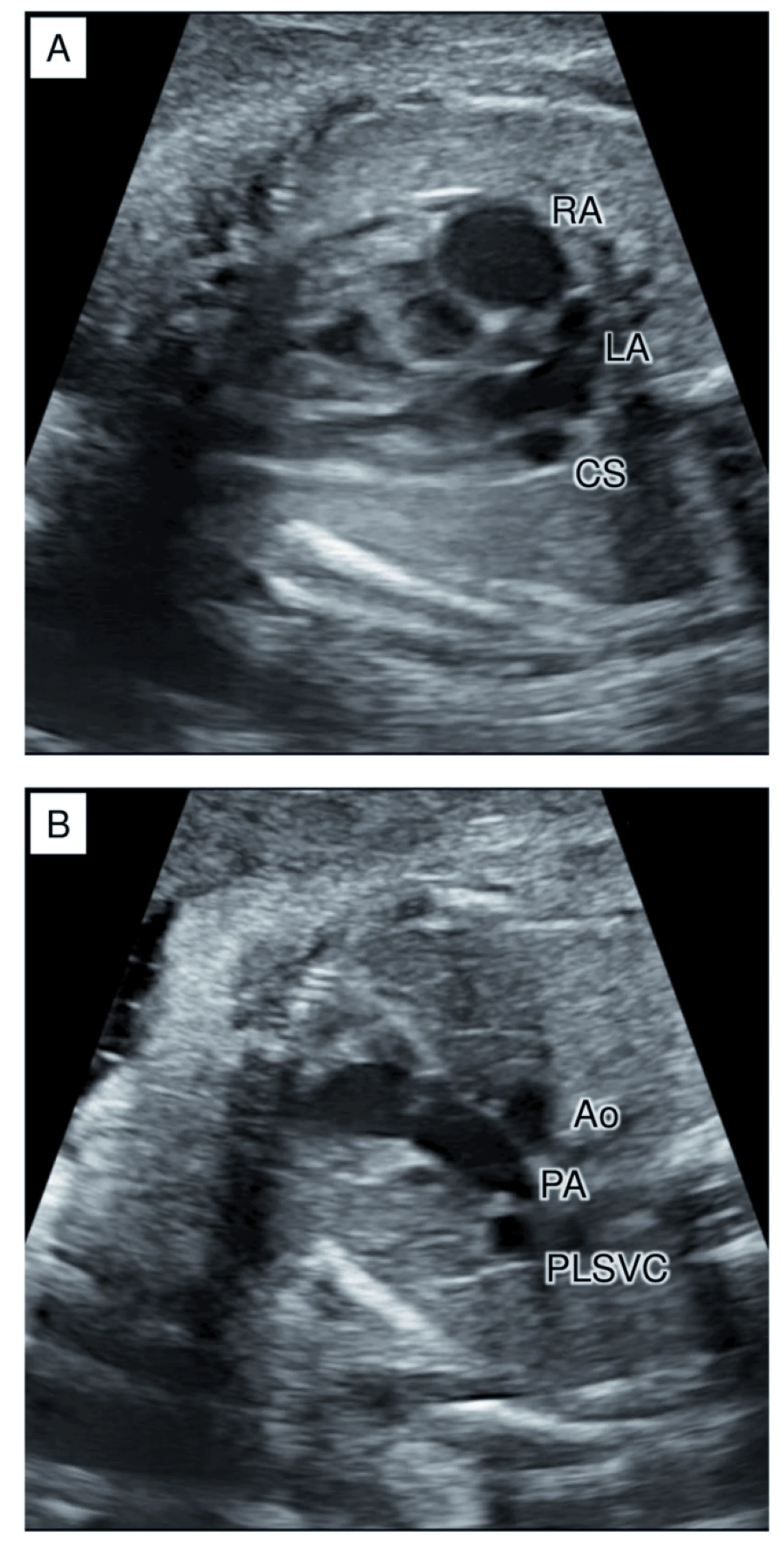
Fetal ultrasonography transitioning from the four-chamber view to the outflow tract and three-vessel views (A) Four-chamber view showing an enlarged coronary sinus adjacent to the left atrium, suggesting the presence of an LSVC. (B) Three-vessel view illustrating the persistent LSVC to the left of the aortic arch and pulmonary artery, with no superior vena cava visible in its typical position on the right. CS: coronary sinus; RA: right atrium; LA: left atrium; Ao: aortic arch; PA: pulmonary artery; LSVC: left superior vena cava; PLSVC: persistent left superior vena cava

**Figure 2:**
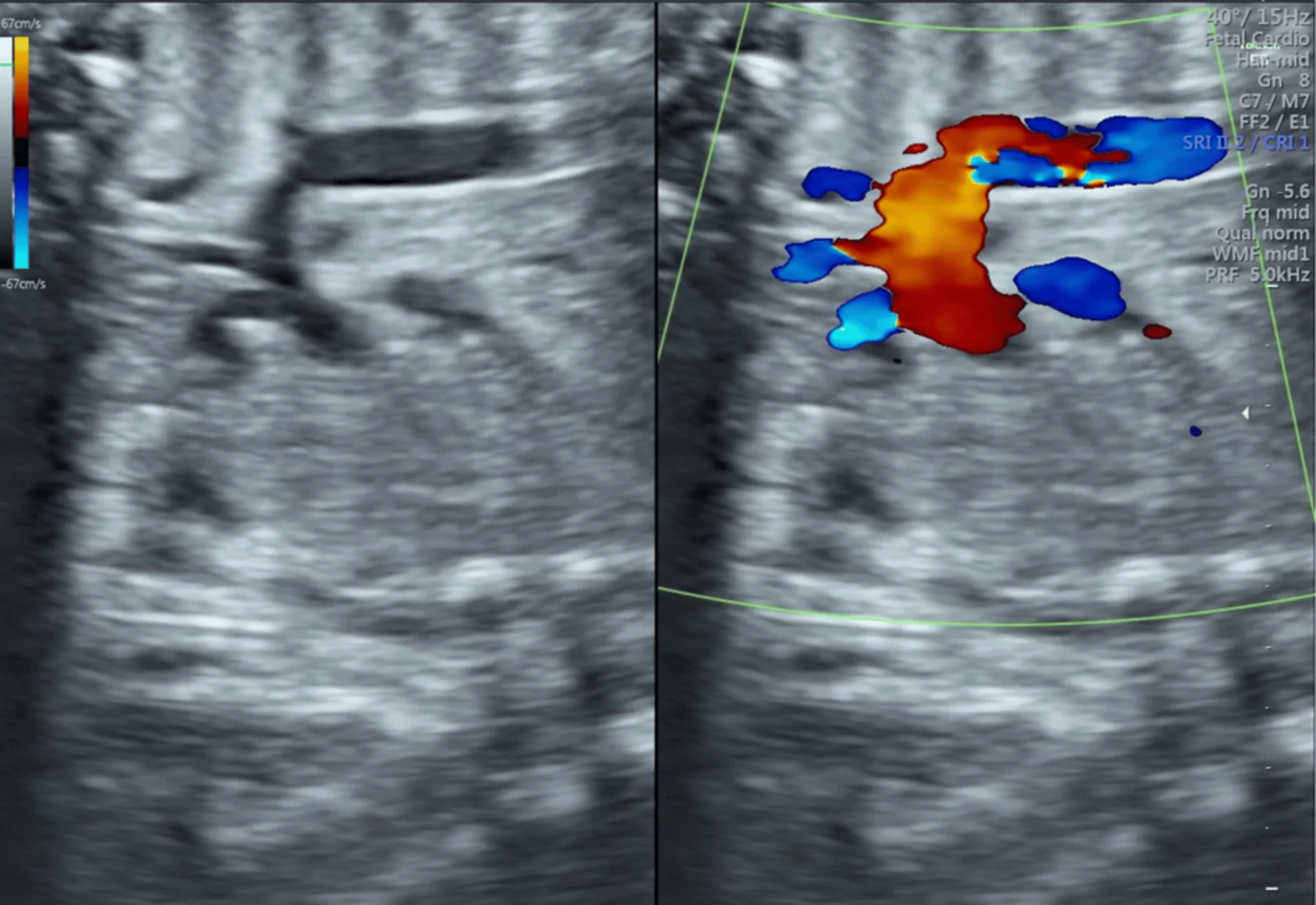
Color Doppler examinations of the aortic arch and aortic isthmus revealed no abnormal flow or significant narrowing

The pregnancy remained uneventful, and at 38 weeks of gestation, the patient underwent a spontaneous vaginal delivery, giving birth to a healthy newborn weighing 3,333 g. Postnatal examination found the infant well-perfused and breathing comfortably, with no murmurs and normal oxygen saturation. Postnatal transthoracic echocardiography on day 2 of life confirmed the presence of a persistent LSVC draining into a dilated coronary sinus, and confirmed the absence of an RSVC. No other cardiac anomalies were seen. These postnatal echocardiography findings correlated with the prenatal diagnosis, definitively establishing an isolated PLSVC with absent RSVC (Figure 3). The newborn remained hemodynamically stable, and no immediate intervention was required. The care team documented the venous anomaly in the medical record for future reference, given its potential relevance for vascular access or other procedures.

**Figure 3:**
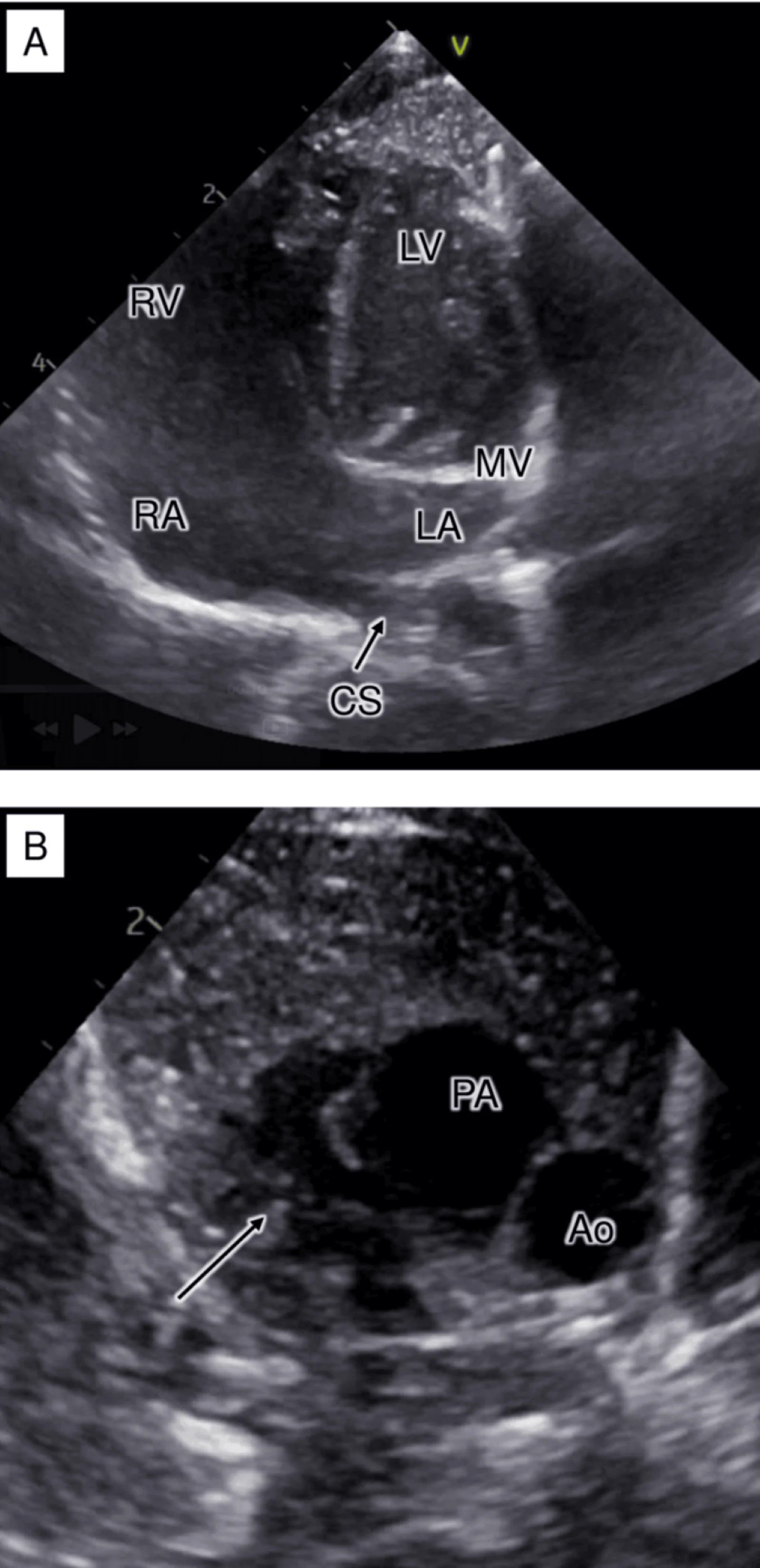
Postnatal transthoracic echocardiography (A) A markedly dilated coronary sinus (arrow) adjacent to the LA. CS inner diameter 5.2 mm, measured at end-diastole (inner edge to inner edge), consistent with drainage from a PLSVC. (B) The arrow marks the expected location of the SVC, which is not visualized, demonstrating the absence of the RSVC. CS: coronary sinus; MV: mitral valve; LV: left ventricle; RV: right ventricle; RA: right atrium; LA: left atrium; Ao: aortic arch; PA: pulmonary artery; RSVC: right superior vena cava; PLSVC: persistent left superior vena cava

## Discussion

This case illustrates a rare vascular anomaly, a PLSVC with an absent RSVC, diagnosed prenatally and confirmed postnatally. Isolated PLSVC without an RSVC is an uncommon finding, especially in an otherwise structurally normal heart. In our case, prenatal detection allowed for careful planning and evaluation of possible associated conditions. We specifically ruled out coarctation of the aorta, which has been reported to co-occur with fetal PLSVC in some instances. Notably, one recent report noted a PLSVC in approximately 21% of fetuses with prenatal coarctation of the aorta, underscoring a strong association [9]. Yonehara et al. also described that the presence of a PLSVC in a fetus may raise suspicion for coarctation, as they reported a case of fetal PLSVC associated with a narrow aortic isthmus that progressed to significant coarctation postnatally [10]. In our patient, however, careful evaluation of the aortic arch via prenatal ultrasound showed a normal-caliber arch and isthmus, with normal flow (Figure 2), and the infant had no evidence of coarctation after birth. This finding emphasizes the need for comprehensive fetal echocardiographic assessment, including Doppler interrogation of the aortic arch, whenever a prenatal PLSVC is identified, so that potential secondary lesions, such as coarctation, are not missed and can be confidently excluded.

Prenatal diagnosis of PLSVC with absent RSVC is unusual but feasible with current imaging techniques. Key fetal echocardiographic views include the 3VV (or three-vessel trachea view), where an isolated PLSVC appears as a single large vessel to the left of the pulmonary artery (with the absence of the usual SVC on the right), and the 4CV, where a dilated coronary sinus can be seen adjacent to the posterior left atrial wall [5,6]. If a PLSVC is found alongside other abnormalities, it could be part of a more complex syndrome (such as heterotaxy or conotruncal cardiac defects), which would affect management and prognosis [1,5]. Fortunately, our patient did not have any such associated issues, consistent with reports that a significant subset of PLSVC cases (around 54% in one series) have no other malformations [3]. Recent evidence indicates that truly isolated PLSVC, with no other cardiac or genetic abnormalities, carries a benign prognosis; Durand et al. reported 100% five-year survival for isolated PLSVC, and lower survival when major cardiac or extracardiac malformations were present [8].

The clinical implications of identifying a PLSVC with absent RSVC extend beyond the prenatal period. Hemodynamically, our infant’s circulation was unaffected, as expected; the LSVC drained into the right atrium via the coronary sinus, maintaining normal venous return. However, knowledge of this venous anatomy is important for future medical or surgical interventions. Central venous access or cardiopulmonary bypass cannulation could be challenging if the practitioner is unaware that the RSVC is absent. Reports in the literature have documented difficulties in placing pacemaker leads or central venous catheters from the right side in patients with an unrecognized absent RSVC, often necessitating a left-sided approach or alternative strategies [3]. In our case, we ensured that the anomaly was clearly communicated to the family and documented for the child’s future healthcare providers. Should the child ever require a central line, pacemaker, or cardiac surgery, awareness of the absent RSVC will allow the team to plan appropriate venous access (usually via the left jugular/subclavian route, or via the hepatic IVC route, if needed) and avoid unexpected complications. Additionally, the markedly dilated coronary sinus that accompanies a PLSVC can be misinterpreted as other pathologies (such as an atrial mass or cyst) if the diagnosis is not known, and it can, in rare cases, impinge on cardiac structures or be associated with arrhythmias [3,11]. Knowing the patient’s venous anatomy thus helps in both interpreting imaging correctly and anticipating any unusual complications.

## Conclusions

In summary, we report a prenatally diagnosed case of an isolated PLSVC with absent RSVC, confirmed postnatally with no associated anomalies. This case adds to the growing evidence that, although PLSVC (with or without an RSVC) is often associated with other congenital abnormalities, it can occur in isolation and have an excellent outcome. Early detection via prenatal ultrasound allowed us to exclude other concerns, such as coarctation of the aorta, and to manage the pregnancy with appropriate expectations. From a practical standpoint, recognition of this venous anomaly is important for clinical care; it alerts clinicians to examine for other potential abnormalities and to take precautions during any invasive procedures. Our patient’s course was uncomplicated, reflecting the generally benign nature of isolated systemic venous anomalies. Ongoing follow-up will include routine pediatric care; no cardiovascular intervention is anticipated. This case underlines the value of detailed fetal echocardiography in identifying vascular variations, improving neonatal preparedness, and documenting such findings to inform future medical management.
